# Demographic and Comorbidities Data Description of Population in Mexico with SARS-CoV-2 Infected Patients(COVID19): An Online Tool Analysis

**DOI:** 10.3390/ijerph17145173

**Published:** 2020-07-17

**Authors:** Carlos E. Galván-Tejada, Laura A. Zanella-Calzada, Karen E. Villagrana-Bañuelos, Arturo Moreno-Báez, Huizilopoztli Luna-García, Jose María Celaya-Padilla, Jorge Issac Galván-Tejada, Hamurabi Gamboa-Rosales

**Affiliations:** 1 Unidad Académica de Ingeniería Eléctrica, Universidad Autónoma de Zacatecas, Jardín Juarez 147, Centro, Zacatecas 98000, Zac, Mexico; kvillagrana@uaz.edu.mx (K.E.V.-B.); morenob20@uaz.edu.mx (A.M.-B.); hlugar@uaz.edu.mx (H.L.-G.); jose.celaya@uaz.edu.mx (J.M.C.-P.); gatejo@uaz.edu.mx (J.I.G.-T.); hamurabigr@uaz.edu.mx (H.G.-R.); 2 LORIA (INRIA, CNRS, Université de Lorraine), Campus Scientifique BP 239, 54506 Nancy, France; laura.zanella-calzada@univ-lorraine.fr

**Keywords:** COVID-19, demographic analysis, comorbidity analysis, web interface development

## Abstract

The Word Health Organization (WHO) declared in March 2020 that we are facing a pandemic designated as COVID-19, which is the acronym of coronavirus disease 2019, caused by a new virus know as severe acute respiratory syndrome coronavirus 2 (SARS-CoV-2). In Mexico, the first cases of COVID-19, was reported by the Secretary of Health on 28 February 2020. More than sixteen thousand cases and more than fifteen thousand deaths have been reported in Mexico, and it continues to rise; therefore, this article proposes two online visualization tools (a web platform) that allow the analysis of demographic data and comorbidities of the Mexican population. The objective of these tools is to provide graphic information, fast and updated, based on dataset obtained directly from National Governments Health Secretary (Secretaría de Salud, SSA) which is daily refreshed with the information related to SARS-CoV-2. To allow a dynamical update and friendly interface, and approach with R-project, a well-known Open Source language and environment for statistical computing and Shiny package, were implemented. The dataset is loaded automatically from the latest version released by the federal government of Mexico. Users can choose to study particular groups determined by gender, entity, type of result (positive, negative, pending outcome) and comorbidity. The image results are plots that can be instantly interpreted and supported by the text summary. This tool, in addition to being a consultation for the general public, is useful in Public Health to facilitate the visualization of the data, allowing its timely interpretation due to the changing nature of COVID-19, it can even be used for decision-making by leaders, for the benefit of the health of the community.

## 1. Introduction

The Word Health Organization (WHO) in January 2020 declared a state of emergency to address the outbreak of a virus in China, a group of cases of “atypical pneumonia” in Wuhan, Hubei province [1], which subsequently it was confirmed that the cause was a new virus, designated as severe acute respiratory syndrome coronavirus 2 (SARS-CoV-2) [2]. The WHO announced that the disease caused by this new coronavirus was a COVID-19, which is the acronym of “coronavirus disease 2019” [3]. Two months after this announcement, the organization issued a statement to alert that the world is facing a pandemic. The cases have appeared in 212 countries, territories and areas; notified in the six WHO regions (America, Europe, Asia Southeast, Eastern Mediterranean, Western Pacific and Africa) [4]. Globally, the numbers of cases due to SARS-CoV-2 continue to increase but exceed 2,954,222 cases confirmed and more than 202,597 deaths [5].

In Mexico, the first cases of COVID-19, were reported by the Secretary of Health on 28 February 2020 [6]. More than sixteen thousand cases and more than fifteen thousand deaths have been reported in Mexico, and it continues to rise [4].

To date, the most common comorbidities associated to COVID-19 found are hypertension (56.6%), obesity (41.7%), and diabetes (33.8%), however there are others that stand out as cancer (6%); cardiovascular disease like coronary artery disease (11.1%) and congestive heart failure (6.9%); chronic respiratory disease like asthma (9%), chronic obstructive pulmonary disease (COPD, 5.4%), obstructive sleep apnea (2.9%); inmunosuppresion as HIV (0.8%), history of solid organ transplant (1%); kidney disease both chronic (5%) and end-staged (3.5%); liver disease like cirrhosis(0.4%), hepatitis B (0.1%), hepatitis C (0.1%); metabolic disease like obesity (BMI ≥ 30) (41.7%), morbid obesity (BMI ≥ 35) (19.0%) [7,8].

According to data from the 2016 National Health and Nutrition Survey (ENSANUT) in Mexico [9] highlights that the 72.5% adult population over 20 years of age is overweight and obese; with hypertension the 25.5%, of which 40% were unaware that they had this disease and only 58.7% of adults with a previous diagnosis were in adequate control (<140/90 mmHg), it also previous diagnosis of this disease is usually higher in women than men (70.5 vs. 48.6%); and with a previous diagnosis of diabetes 9.4%. The main causes of death in adults are due to heart disease, followed by diabetes, malignant tumors, liver disease, and accidents. In men, heart disease represents 20.1% of mortality, followed by diabetes with 14.1%; while in women heart disease represents 22.7% and diabetes 18.6% [10].

Public Health actions play an important role, allowing communities that anticipate the virus to learn and adapt from action plans that have proven to be effective. Although there are non-modifiable factors that could explain the success or failure, to contain the disease, therefore, it is necessary to investigate the behavior of diseases, identify the particular characteristics (demographic, comorbidities, among others) of each region and thus have tools to confront diseases with greater precision.

To support this current situation, there are some tools, available online, that allow the visualization of COVID-19 cases, such as the case of John Hopkins University [11], which created an interactive map, to monitor coronavirus cases in the world. in real time, which shows the confirmed cases and deaths worldwide, by each country, and also by each of the United States of America (positive cases, deaths, cases tested, and accumulated hospitalizations for the disease), presents three graphs, which representatively show the confirmed cases, logarithmic curve and the accumulated daily cases, respectively. Another tool that stands out in the network belongs to the company Google [12], which shows information on confirmed cases, cases per million people, people recovered, deaths, both globally, by country and in some of them by state. It contains an interactive map that when you hover over the desired region, the summary with the aforementioned characteristics is displayed, a histogram is included which contains the cases over time, according to the selected region.

However, until now there is no tool that describes the special characteristics of the Mexican population, therefore, this article proposes two online visualization tools (a web platform) allow the analysis of demographic data as well as comorbidities, present in patients of the Mexican population. The objective of these tools is to provide rapid graphical information based on the data obtained by the Mexican Ministry of Public Health and to see how it evolves in the different demographic groups as well as to have the risk factors in these groups that aggregate the reported comorbidities in different countries. Therefore, in a condensed way, the aim of the present work is to provide an open access, easy to manipulate, and intuitive graphic tool that allows the visualization to identify the behavior of the disease, according to the characteristics selected by the user, thus allowing the international comparison of particular data and providing the evolution of the disease. In addition to providing a tool for the scientific environment without the need for the experience or learning curve in the manipulation or visualization of data.

This paper is organized as follows. Section 2 presents a detailed description of the development of this visualization tool, as well as the methods applied to develop different proposed plots. Section 3 presents the plots and the interface of the proposed tools using positive SARS-CoV2 patients possible data that is useful in Public Health. In Section 5, a discussion about the development and use of the proposed tools and conclusions of the present work.

## 2. Materials and Methods

In this section is described in detail dataset and features, which describe the confirmed, suspicious, and negatives patients to SARS-CoV-2. Followed by the description of the development of the online tool.

**Figure 1:**
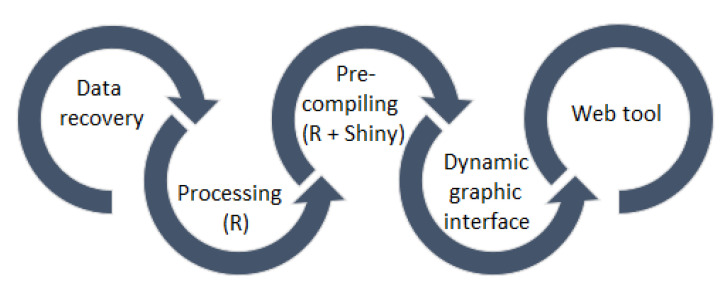
Flowchart of the methodology proposed.

A flowchart of the methodology followed is presented in Figure 1. Initially, the data is recovered from the official site of the National Governments Health Secretary of Mexico. A processing to these data is applied using the software R and, subsequently, a pre-compilation is performed the R package, Shiny. Then, the dynamic graphic interface is developed based on the previous steps and, finally, the web tool is obtained.

### 2.1. Database Acquisition

Dataset is obtained directly from National Governments Health Secretary (Secretaría de Salud, SSA https://www.gob.mx/salud/documentos/datos-abiertos-152127) which is daily refreshed with the information related to SARS-CoV-2 infected patients and suspicious of infection as well as confirmed like Non-SARS-CoV-2 infected patients but with other severe acute respiratory syndrome or similar (like influenza H1N1, or respiratory syncytial virus among others). This dataset is comprised by 18 features listed in Table 1 which include most common comorbidities reported around the world related to COVID-19 [13].

This analysis favored comorbidities and demographic features, i.e. diagnosed diseases as well as the characteristics of entity, gender and age. Additional information of features are available together with the dataset.

### 2.2. Database Pre-Processing

This data set is provided individually for each medical unit in Mexico, and standardized by federal government guidelines. In the case of entity of patients only state entity is used, the municipality feature in Mexico implies a granularity of 2502 entities, and in the case of birth entity, in this study is discarded given that the aim is a study of the living location of the infected and potentially infected patients. This last restriction is also applied to nationality, country of origin and if the patient is a migrant.

### 2.3. Web Interface Development

To allow a dynamical update and friendly interface, and approach with R-project, a well-known Open Source language and environment for statistical computing [14] and Shiny package [15], were implemented. The dataset is loaded automatically from the latest version released by the federal government of Mexico. Users can choose to study particular groups determined by sex, entity, type of result (positive, negative, pending outcome) and comorbidity. Main section of the interface shows the analysis, statistical summary and figures related to the sub-dataset extracted with the selected determinants of the group. These data could be refreshed instantly changing any of the determinants mentioned before. The image results are plots that can be instantly interpreted and supported by the text summary.

### 2.4. Demographic Analysis

For the demographic analysis, a selection among a series of parameters concerning the demography of the population that have been present in patients with a positive, negative or uncertain SARS-CoV-2 result. With this tool it is possible to develop an analysis based on the relation between the demography and the cases of COVID-19, based on the time, the age and the sex. The different graphs generated to carry out the analysis are described below.

#### 2.4.1. Histogram of the Distribution

A histogram is represented as a plot that underlay the frequency distribution of a set of continuous data. Each frequency value in a histogram is represented with a bin, which is a bar that gives information of the frequency of each sample. The main purpose of histograms is the provide a tool for the inspection of the data based on their distribution, outliers, skewness, among other characteristics.

Therefore, for the visualization and analysis of the different demographic features concerning the Mexican population in relation to COVID-19, histograms are constructed. This tool allows to do a risk assessment and diagnosis through the acquisition of an overview of the current situation in relation to this disease (based on the update given by the federal government of Mexico), making it possible to apply filters to the information shown in the histogram. These filters allow to make a selection in the demographics of the population that want to be taken into account for the construction of the histogram. The demographic options that can be chosen concern sex and territorial region [16].

#### 2.4.2. Boxplot

A boxplot can be described as a visualization tool that presents a summary of a given dataset, including the median, the inter quartil range and the outer range. The median value represents the middle data observation of a ranked dataset, giving also the central tendency of the data and the 50 percentil. The inter quartil range represents 50% of the ranked data and it is measured from the lower quartil value (25 percentil) to the upper quartil value (75 percentil). Finally, the outer range concerns the whiskers, and they are represented from the lower and upper quartil values to the minimum and maximum values of the data, respectively [17].

Therefore, the construction of a boxplot is proposed since it represents a suitable method for analysis, allowing to know the trend and skewness of the Mexican population for the different options of demography included.

#### 2.4.3. Daily Evolution Plot

A daily evolution plot is another visualization tool that allows to model the behavior of a variable with respect to time. This plot allows to have a global vision of the changes that have been shown in this variable in a period, allowing to analyze the way in which it has behaved and to be able to generate hypotheses about future behavior based on the past.

In this way, the construction of a daily evolution plot based on the behavior of the positive cases of SARS-CoV-2 presented in Mexico gives a general understanding of how the disease has evolved depending on the different demographic options provided. This plot allows to know how the cases have increased or decreased over time, to observe peaks and valleys, and to see the trend that the spread of COVID-19 follows.

#### 2.4.4. Density Plot

A density plot is another visualization tool that gives information of the distribution of data over a continuous interval. This plot is a variation of the histogram that applies kernel smoothing to plot the values, providing smoother distributions by smoothing the noise. However, one advantage of these plots over histograms is that density plots determine better the distribution shape because they are not influenced by the number of bins used. The peaks presented in a density plot provide information of how the values are concentrated over the interval [18].

Based on this plot, it is possible to know the distribution of the population that has presented COVID-19 from a slightly different approach to that obtained with the histogram. In this way, information can be obtained on where the highest population density is found with the presence of this disease and also where the lowest density is, based on demography.

#### 2.4.5. Incidence Plot

Incidence will be understood as the number of new cases, through time [19,20]. A cumulative incidence plot shows the proportion of incidence of subjects with a particular condition over time. The curves presented in this graph disclose the behavior of the proportion of these subjects [21].

This plot reveals the behavior of the incidence of patients who have tested positive for the SARS-CoV-2 with respect to time. In this way it is possible to analyze how the incidence of this disease has changed, the increases and decreases, and thus provide tools for evaluating the action taken towards the disease. For example, evaluate the measures that have been taken to prevent this virus from spreading further and thus decrease the incidence. In particular this plot presents a regression of log-incidence over the time, with a confidence interval (2.5–97.5%).

### 2.5. Comorbidity Analysis

For the comorbidity analysis, it is also possible to make a selection among a series of comorbidities that have been present in patients with a positive, negative or uncertain SARS-CoV-2 result. These comorbidities are high impact diseases in Mexico. Therefore, it is impossible to make a correlational analysis in which the condition of comorbidities can be seen in relation to the different demographic options provided.

#### 2.5.1. Comorbidity Overlapped Histogram

The comorbidity overlapped histogram is a visualization and analysis tool that allows to compare the population with a positive, negative or uncertain result of SARS-CoV-2 that present comorbidities and those who do not present, based on the age. With this graph, a risk assessment can be made by analyzing the frequency with which comorbidities occurred or not within the selected population. This frequency is based on age ranges. Therefore, it is possible to have an overview of the frequency with which comorbidities occur or not, in relation to people who have COVID-19 and those who do not.

#### 2.5.2. Double Boxplot

The construction of this graph that shows two boxplots, where each one refers to presenting comorbidities or not presenting them, allowing to observe allowing to know the trend and skewness of the Mexican population for the different options of demography included and letting know if there are outliers or those that come out of the age ranges that are considered within the total sample. With this tool it is possible to know which is the median and quartiles of the population sample according to age, allowing a comparison of the risk between the two groups of subjects, according to the age intervals.

#### 2.5.3. Comorbidity Incidence

This cumulative incidence plot, shows the proportion of incidence of patients divided by gender and with at least one comorbidity over time. This graph allows to observe the behavior along the time of the impact of the comorbidities and how this increase and promote the SARS-CoV2 infection.

## 3. Development and Results

**Figure 2:**
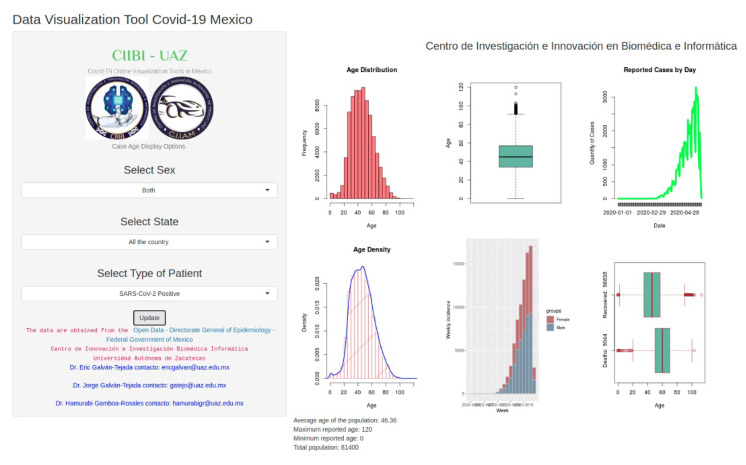
Layer of the web interface that allows to analyze the behavior of COVID-19 based on demographic information.

**Figure 3:**
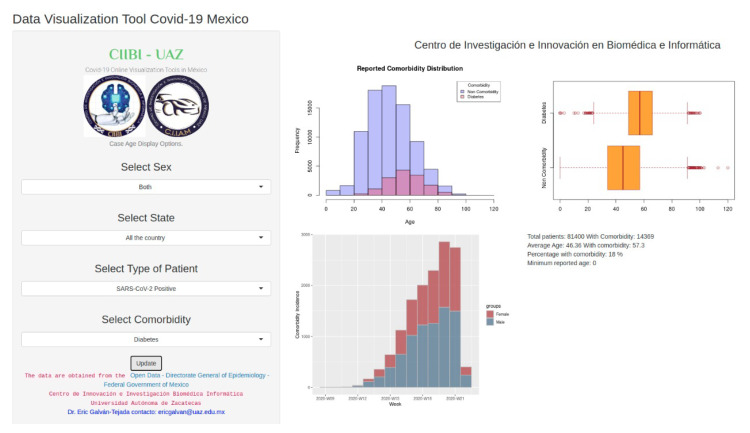
Layer of the web interface that allows to analyze the behavior of COVID-19 based on comorbidity information.

A main web interface to study demographic and comorbidity, is comprised by two layers, shown in Figure 2 and Figure 3, respectively. The main web interface can be accessed in http://covid19.ciibi.mx/, and in particular demographic tool from http://covid19demographic.ciibi.mx/ and comorbidity interface from http://covid19comorbidity.ciibi.mx/.

In the left side of the layers is presented a series of drop-down lists giving the option to choose the different parameters to take into account for the generation of the graphs, which are presented on the right side. In the layer that allows the demographic analysis (Figure 2), it is possible to make a selection of parameters within the options that concern the sex, the state (region) of the country and the type of patient (positive or negative result to SARS-CoV-2 or uncertain ). While the layer corresponding to the analysis of correlation with comorbidities (Figure 3), allows to make a selection of parameters within the options that concern sex, the state (region) of the country, the type of patient (positive or negative result to SARS-CoV-2 or uncertain) and comorbidity.

### 3.1. Demographic Analysis Tool

**Figure 4:**
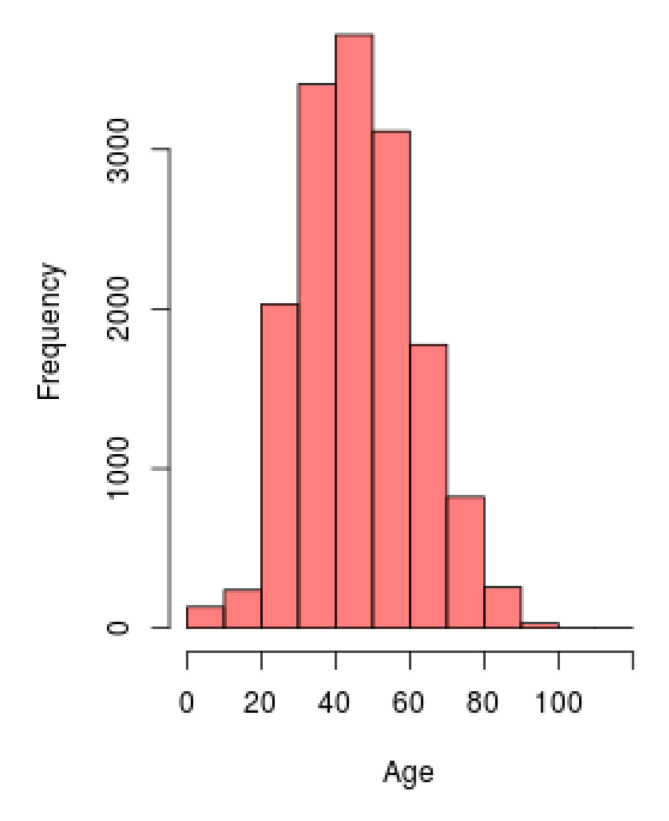
Histogram of the distribution by age the population from all the territory with positive SARS-CoV-2.

Figure 4 shows an example of the histogram constructed in the web interface, using the layer for demography analysis. The information used for the development of this graph concerns to the distribution of the population, including both sexes, from the all the territory that obtained a positive result of SARS-CoV-2, according to their age. Each bin of the graph represents the frequency of subjects with presence of COVID-19 for each age range.

**Figure 5:**
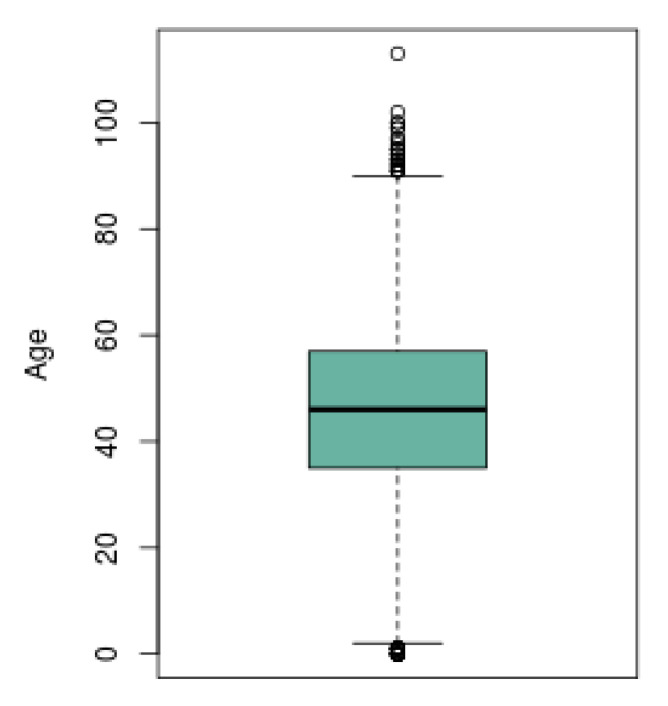
Boxplot of the distribution by age the population from all the territory with positive SARS-CoV-2.

In Figure 5 is presented the boxplot obtained from the same subjects sample selected as in the previous graph. The black line in the middle of the box representes the median age of the population with presence of COVID-19. The horizontal edges of the box represent the lower and upper quartiles, and the short lines that are at the ends of the projecting lines of the boxes represent the minimum and maximum age presented by the subjects. The small circles that exceed the maximum and minimum limits represent the outliers, which are the cases of the subjects that presented an atypical age among the population with the disease.

**Figure 6:**
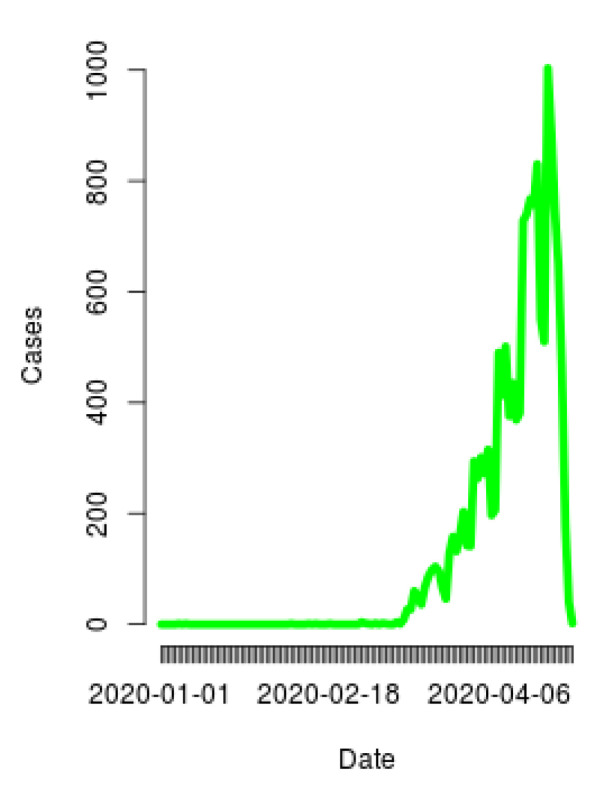
Daily evolution plot of cases presented in the population from all the territory.

Figure 6 shows a graph of the daily evolution of cases of COVID-19 in the population from all the territory, where it is possible to observe the behavior of the increasing or decreasing of cases per day.

**Figure 7:**
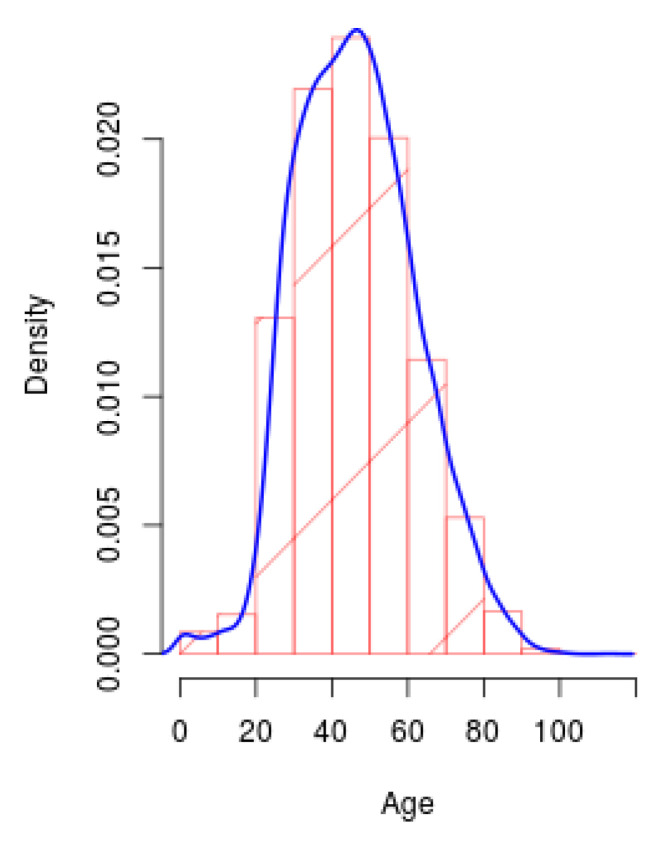
Density plot of the population from all the territory with positive SARS-CoV-2.

Figure 7 shows the density plot of the population with presence of COVID-19 from all the territory. The red bins represent the number of subjects for each age range, while the blue line represents the envelope of the bins. The envelope allows to see in a smooth way the distribution of the subjects according to age.

**Figure 8:**
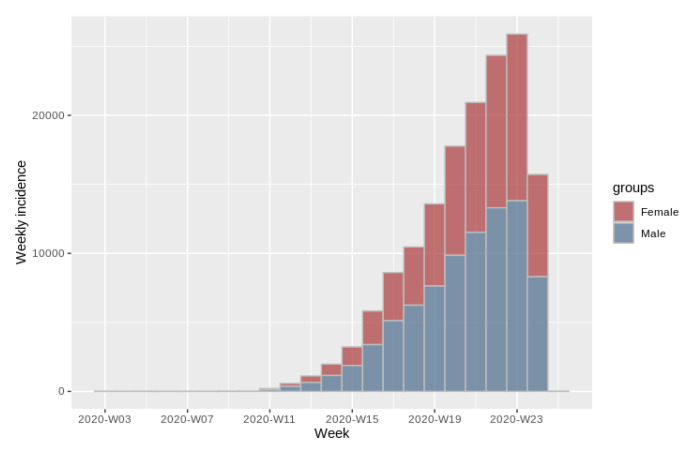
Weekly incidence plot of cases of SARS-CoV-2 in the population from all the territory.

Figure 8 shows a graph of the weekly incidence of COVID-19 among the population from all the territory. Bins in red represent the incidence of females, while bins in blue represent the incidence of males. With this graph it is possible to observe the behavior of the incidence of the disease throughout the weeks and to make a comparison of this incidence between both sexes.

**Figure 9:**
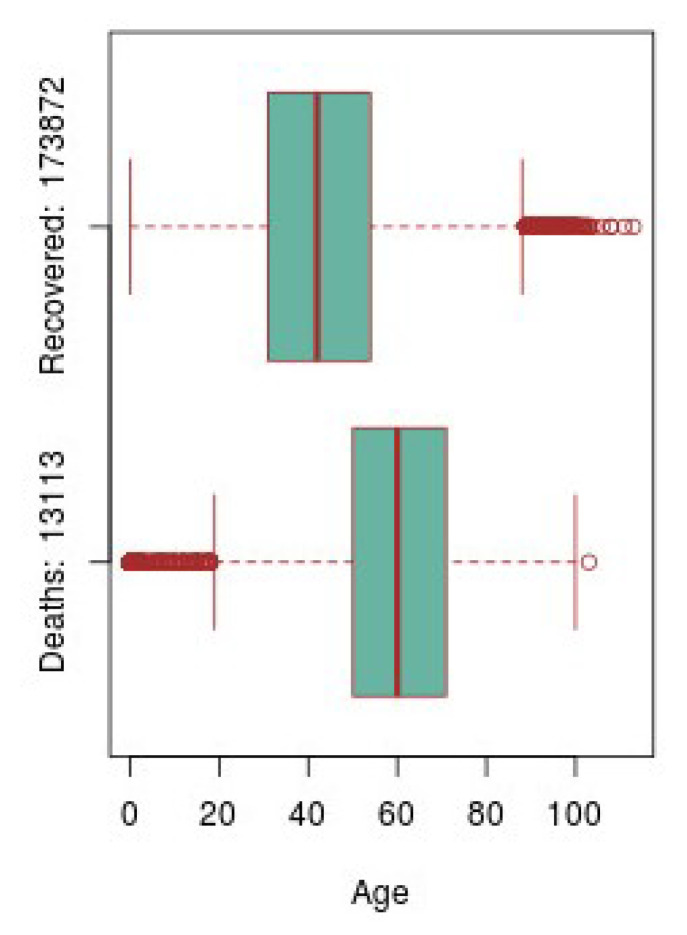
Double Age boxplot comparison of recovered cases against deaths.

Figure 9 shows two boxplots, one referring to the number of patients recovered from COVID-19 and the other to the number of cases of patients who died due to this disease. With these graphs it is possible to make a direct comparison between the total number of patients who are recovered against the deceased, taking into account the age ranges of the patients. In addition, it is also possible to compare the distribution of these numbers, the mean, the quartiles, and those outliers that have occurred.

### 3.2. Comorbidity Analysis Tool

**Figure 10:**
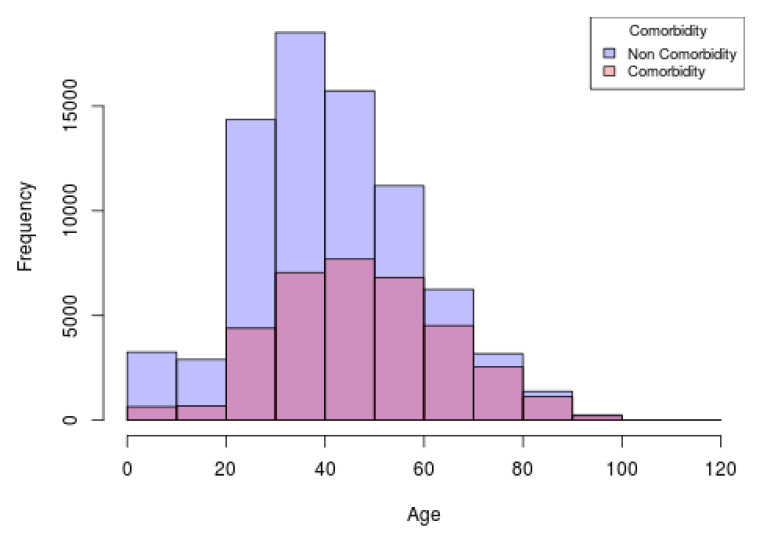
Comorbidity overlapped histogram of the population from all the territory with positive SARS-CoV-2.

Figure 10 shows an example of an histogram constructed in the web interface, in the layer of comorbidity. The information used for the development of this graph concerns to the comorbidity distribution of the population from the all the territory that obtained a positive result of SARS-CoV-2, including both sexes, that presented at least one comobirdity. Each bin of the graph represents the frequency of subjects with comorbidities (shown in pink) and with non comorbidities (shown in purple) for each age range.

**Figure 11:**
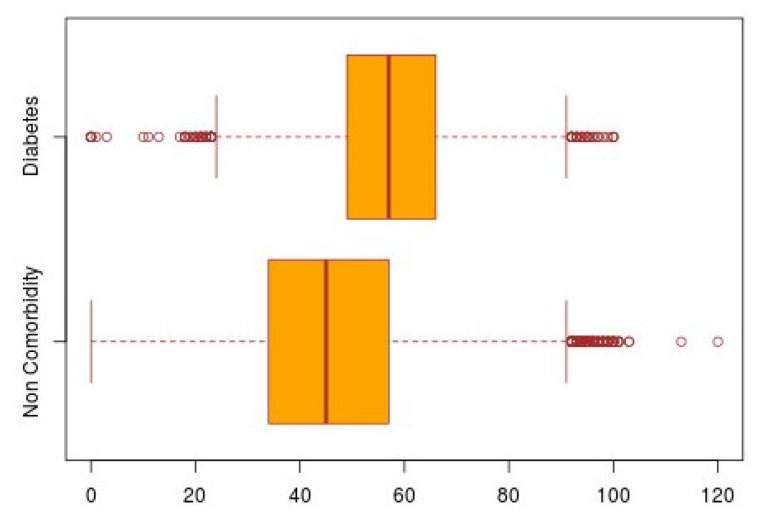
Double boxplot of the population from all the territory with positive SARS-CoV-2 with/without diabetes comorbidity.

An example of a double boxplot is shown in Figure 11. For the construction of this graph, the same parameters from the previous overlapped histogram are applied. The boxes are developed based on the age of the subjects. The red lines that are in the middle of the boxes represent the median value, which is to say, the median age of the subjects, while the vertical edges represent the lower and upper quartiles. The vertical short lines that are at the ends of the projecting lines of the boxes represent the minimum and maximum age presented by the subjects. The small circles that exceed the maximum and minimum limits represent the outliers, which are the atypical samples of the subjects, that is, those rare cases in which the subjects with presence of comorbidities had ages that did not fall within the age range of the population.

**Figure 12:**
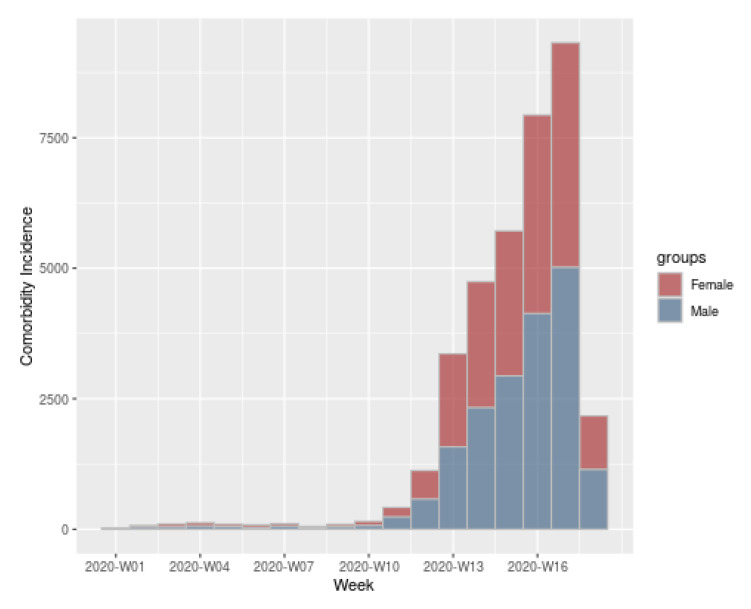
Comorbidity incidence of the population from all the territory with positive SARS-CoV-2.

In Figure 12 is shown an example of an histogram representing the comorbidity incidence of the same sample as the used in the previous graphs. In this graph it is possible to observe the incidence among the weeks of COVID-19 presented by females (shown in red) and males (shown in blue) with at least one comorbidity.

## 4. Applications in Public Health

Public Health is an empiric and multidisciplinary field whose goal is to assure conditions in which people can be healthy [22], seeking the health of the community.

The use of statistical tools, of course working together with the scientific method applied in research in the area of health, gives support to adopt recommendations in treatment, diagnosis or decision-making for the benefit of society, the latter is of special interest to public health, since it carries out projects, proposals and strategies to improve the health of populations [23].

The analysis of the information in an objective and structured way is vital for the development of actions in Public Health, the proposed tool allows segmenting the population by different characteristics, which allows viewing groups with similarities and/or patterns, with greater or lesser risks [24].

With the tool described in this paper, data is displayed graphically and and subgroups with very specific characteristics are quickly identified, for example: Until 27 April, in Mexico, 71,103 people have been studied in search of SARS-CoV-2, with a median age of 41.53 years, with a distribution by sex of 35,507 women with a median age of 40.95 years; and 35,596 men with an average age of 42.11 years.

Of the patients studied to date, there are 15,529 positive cases for SARS-CoV-2 with an average age at the time of diagnosis of 46.55 years of age; the female population corresponds to 6552 positive cases, with a median age of 45.86 years; for the male population, 8977 cases of the disease were detected, with a median age of 47.05 years.

This indicates that the disease predominates in male gender, exceeding 15.61% and also the average age of infection is higher with 1.19 years versus female gender.

The above is an example of how the platform statistics can be interpreted, providing alternatives in the query of statistical data of COVID-19 in the country, for example the histograms shown allow to quickly identify the age groups with more or less affectation, the box diagram allows to identify the range of affected ages, among other graphical data shown.

It is possible to streamline the analysis of information by researchers and the general public, with the use of this tool and other tools can be synergized to determine the usefulness of the preventive measures adopted to contain the disease, and thus allow us to recognize if it is necessary to propose new strategies to mitigate the undesirable effects.

The success of Public Health depends on compliance with the basic rules of equity, associations and social justice, as well as the mobilization of local, national and international resources [22]. Thanks to the analysis and interpretation of the data, in this health case, the rulers can take the most pertinent path, or simply to keep the general population informed, and motivate scientific research.

## 5. Discussion and Conclusions

To our knowledge, this is the first free online tool to analyze the demographic and comorbidities characteristics for the Mexican population related to COVID-19. The current available tools presenting similar approaches are international projects [11,12], none of them focusing on Mexico, and they have not yet linked cases of COVID-19 to comorbidities, nor has age been taken into account for the analysis of the disease, which are some of the contributions of this work.

This analysis tool is presented in two parts, one part is focused on the analysis of COVID-19 in relation to the demography, and the other part is focused on the analysis in relation to a set of comorbidities, taken into account demography too. For the development of this work, the database provided by the federal government of Mexico through its public health secretary is used. This data is being updated according to the country’s government. Based on this information, the main purpose of the approach presented in this work is to provide a series of tools for visualization and analysis of the data containing information about the development of COVID-19 in the country. These tools allow to extract in a fast, reliable, and accessible way this information from the population with a positive, negative and uncertaing result to the test of this disease.

It is important to mention that there are other local tools that can be used to perform the proposed analysis, like R-Project and Python, among others. However, these tools require expertise to develop accurate plots and datasets management, which are the core to extract information from data. One of the advantages of the approach proposed here is that, given the current spreed of the SARS-CoV-2 virus, time becomes an important variable to consider in order to develop research around this topic, and the tools implemented here are based on it, allowing to know the evolution of the disease in a user-friendly way. While this point can make using the tools mentioned above a little more complicated if the user is not familiar with those environments.

Another important advantage of this work is that, being user-friendly, it allows the majority of the population to easily manage the selection of parameters for the display of information. In addition to that, when generating the display, it shows the results in a graphical and simple way allowing the interpretation of the results to be easy. This is an important point because in this way it is easier to keep the population information on the current situation and also, it allows an own analysis to be made, eliminating the bias that could be included when someone relies on the analysis of a third party to make an own judgment.

Therefore, it is possible to conclude that these online tools are an important contribution to accelerate and facilitate the evaluation of the effect of COVID-19 on the Mexican population, with updated data according to the Mexican government, in addition to being easy to use by users, accessing to different subgroups of characteristics quickly, observing possible behaviors in similar populations and allowing the evaluation or implementation actions for the benefit of community health.

However, among the limitations of this work, it can be mentioned that the updating of the information displayed depends entirely on the data provided by the government, since they are the official data registered by the country, so, in the case that the updating of this data presents any inconvenience, the tool we present will reflect this. In addition, since the access to data depends on what the government provides, so it is not possible to generate an analysis with our tool for all the states of Mexico, since it is not possible to access the data of some of them. Another limitation is that, currently, our proposal is applied focusing nationally, so it does not allow its use for countries other than Mexico.

Based on this, as future work, these tools can be fed with information from a greater number of states in the Mexican Republic, hospitalized cases, recovered cases, and deaths. Also, it is proposed to broaden the focus of the work and allow it to have international access, giving the option that the data with which the tool is fed is provided by the user and thus does not depend on the data provided by a third party. Finally, this tool is updated every day with the mentioned tools but could be increased with more plots and data interpretations.

The role that leaders play is crucial in the world and of course in Mexico; the pandemic that we face represents a challenge both now and in the future, in many disciplines, but undoubtedly today Public Health represents the crucial axis to better cope with this situation, all the decisions made, impact on the community and in the search for the greatest benefit in terms of health, the use of tools such as the one proposed, allow for agile analysis by experts so that there is greater effectiveness and efficiency of public policies. 

## Figures and Tables

**Table 1:** Table of the features included in the dataset.

Feature	Description	Posible Values
Update Date	Dataset is complemented daily, this features allos the identification of the last refresh	Date format YYYY-MM-DD
Id	Identification of the case number	Numeric Integer
Origin	To comprise this data set is used a sentinel surveillance methodology of the system of respiratory disease monitoring health units (RDMHU). The RDMHU include medical units of the first, second or third level of care and also participate as RDMHU the third level units that, due to their characteristics, contribute to widen the panorama of epidemiological information, among them those with specialties in pneumology, infectious diseases or pediatrics.	1—RDMHU 2—Out of a RDMHU 99—Unknown
Sector	Type of national system institution which give primary atention.	
Medical Unit	Id of the medical unit who give primary atention	
Sex	Patients sex	1 Female 2 Male
National entity	Patients birth entity	Numeric Integer of entity ID
Local entity	Patients residence entity	Numeric Integer of entity ID
Municipality	Patients residence minucipality	Numeric Integer of municipality ID
Patient Type	Type of care the patient received in the unit. It is called outpatient if returned home or inpatient if admitted to hospital.	1—Outpatient 2—Hospitalized 3—Unknown
Entry date	Date of admission of the patient to the care unit.	Date format YYYY-MM-DD
Date of symptoms	Date the patient’s symptomatology began.	Date format YYYY-MM-DD
Date of death	Date the patient died.	Date format YYYY-MM-DD
Intubated	Patient required intubation.	Yes/No
Pneumonia	Patient was diagnosed with pneumonia.	Yes/No
Age	Age in years	Numeric Integer
Nationality	Identifies if the patient is Mexican or foreign.	1—Mexican 2—Other 3—Unknown
Pregnancy	Identify if the patient is pregnant.	Yes/No
Speak Native language	Identify if the patient speaks indigenous language.	Yes/No
Diabetes	Diagnosis of diabetes.	Yes/No
COPD	Diagnosis of Chronic obstructive pulmonary disease.	Yes/No
Asthma	Diagnosis of Asthma	Yes/No
immunosuppressed	Patient has immunosuppression.	Yes/No
hypertension	Patient has hypertension	Yes/No
other comorbidity	Other diseases	Yes/No
Cardiovascular	Diagnosis of cardiovascular disease.	Yes/No
Obesity	Patient has obesity	Yes/No
Chronic kidney	Diagnosis of chronic renal failure.	Yes/No
Smoking	Patient has a smoking habit.	Yes/No
Other case	Patient had contact with another case diagnosed with SARS CoV-2	Yes/No
Result	Identifies the sample analysis result reported by the National Network of Epidemiological Surveillance Laboratories.	1 Positive SARS-CoV-2 2 Non-positive SARS-CoV-2 3 Outstanding result
Migrant	Identify if the patient is a migrant.	Yes/No
Nationality	Identify the patients nationality.	Name of the Country
Country of origin	Country from which the patient left for Mexico.	Name of the Country
UCI	Identifies if the patient required admission to an Intensive Care Unit.	Yes/No
